# Synergistic cytotoxicity effect of the combination of chitosan nanoencapsulated imatinib mesylate and quercetin in BCR-ABL positive K562 cells

**DOI:** 10.22038/IJBMS.2023.68472.14934

**Published:** 2023-03

**Authors:** Rohollah Kamyabi, Alireza Jahandideh, Negar Panahi, Samad Muhammadnejad

**Affiliations:** 1 Department of Veterinary Basic Sciences, Science and Research Branch, Islamic Azad University, Tehran, Iran; 2 Department of Veterinary Surgery, Science and Research Branch, Islamic Azad University, Tehran, Iran; 3 Gene Therapy Research Center, Digestive Diseases Research Institute, Tehran University of Medical Sciences, Tehran, Iran

**Keywords:** Chitosan, Chronic myeloid leukemia, Imatinib, K562, Quercetin

## Abstract

**Objective(s):**

Intolerable side effects and resistance to chemotherapeutic drugs have encouraged scientists to develop new methods of drug combinations with fewer complications. This study aimed to investigate the synergistic effects of quercetin and imatinib encapsulated in chitosan nanoparticles on cytotoxicity, apoptosis, and cell growth of the K562 cell line.

**Materials and Methods:**

Imatinib and quercetin were encapsulated in chitosan nanoparticles and their physical properties were determined using standard methods and SEM microscope images. BCR-ABL positive K562 cells were cultured in a cell culture medium, cytotoxicity of drugs was determined by MTT assay and the effects of nano drugs on apoptosis in cells were investigated by Annexin V-FITC staining. The expression level of genes associated with apoptosis in cells was measured by real-time PCR.

**Results:**

The IC_50_ for the combination of the nano drugs at 24 and 48 hr was 9.324 and 10.86 μg/ml, respectively. The data indicated that the encapsulated form of drugs induced apoptosis more effectively than the free form (*P*<0.05). Moreover, the synergistic effect of nano drugs in statistical analysis was proved (*P*=0.001). The combination of nano drugs resulted in the caspase 3, 8, and TP53 genes upregulation (*P*=0.001).

**Conclusion:**

The results of the present study showed that the encapsulated form of imatinib and quercetin nano drugs with chitosan has more cytotoxicity than the free form of the drugs. In addition, the combination of imatinib and quercetin as a nano-drug complex has a synergistic effect on the induction of apoptosis in imatinib-resistant K562 cells.

## Introduction

Today, despite significant advances in cancer treatment, it is still one of the leading causes of death worldwide. Leukemia is a group of cancers that usually start in the hematopoietic stem cells in the bone marrow and cause the formation of a large number of abnormal white blood cells (1). Chronic myeloid leukemia (CML) is a myeloproliferative disease that results from the proliferation of multi-potential stem cells. CML is a malignancy that is usually related to an acquired genetic abnormality called the Philadelphia chromosome (2). This abnormality is present in more than 90% of patients and is the result of rearrangement between the *BCR* and Abelson genes (3). BCR-ABL oncoprotein interacts with other cytoplasmic proteins to activate the Ras-extracellular signal-regulated kinase (Ras-ERK), phosphatidylinositol 3-kinase (PI3K)/protein kinase B (AKT), PI3K-Akt, and signal transducer and activator of transcription 5 (STAT5) that lead to proliferation and resistance to apoptosis in cancer cells (4). Except for CML cases recently diagnosed during pregnancy, the first treatment for this condition is the use of a class of tyrosine kinase (TKI) inhibitors (5). Today, dasatinib, bosutinib, nilotinib, and imatinib are among the tyrosine kinase drugs approved by the US Food and Drug Administration (FDA) and the European Medicines Agency (EMA). Imatinib is one of the most significant TKIs that binds especially to Abl, inhibits substrate phosphorylation, and interrupts the signal transduction pathway that leads to cancer cell apoptosis (6). One of the most essential problems in the treatment of CML is the resistance of patients to imatinib, the mechanisms of which have been investigated in many pieces of research (7, 8).

Synergistic therapy can lower the dose of drug agents and reduce drug resistance and therapeutic side effects in patients. Studies have displayed that mixing anticancer drugs with other anticancer agents with various mechanisms of action can rise their synergistic effect and lessen their resistance (9-11). The combination of platinum-based anticancer drugs (12), proteasome inhibitors (13), aminopeptidase inhibitors (14), cyclooxygenase-2 inhibitors (15), and histone deacetylase inhibitors (16) with imatinib has been studied so far.

Flavonoids are phytochemical compounds with diverse medicinal outcomes that have been considered by scientists in recent years. Quercetin (3,3’,4’,5,7-pentahydroxyflavone) is a natural pigment and a type of flavonoid found in different fruits and vegetables (17). This element is one of the most important antioxidants needed by the human body (18). Studies demonstrate that combining quercetin with other drugs may increase its anti-proliferative and apoptotic effects and reduce inflammation by preventing the secretion of proinflammatory agents such as interleukin-1β from monocytes and tumor necrosis factor (TNF) (19). Investigations on this agent have shown that quercetin has anti-cancer effects due to its ability to reduce proliferation, induce apoptosis, induce cell cycle arrest and inhibit mitotic processes by modulating cyclins (20, 21). It could also activate proapoptotic molecular pathways, including PI3K/Akt/mTOR and mitogen-activated protein kinases (MAPK) (20, 21). Other studies have shown that quercetin inhibits the Wnt/β-catenin signaling pathway, which may be involved in its antiproliferative effect (22). In addition, studies have revealed that quercetin can raise the drug’s effectiveness by decreasing the expression of proteins associated with multidrug resistance and anti-apoptotic resistance. So far, the synergistic effects of quercetin in combination with various anticancer drugs in different categories of human cancer cells, such as MCF-7, SMMC-7721, and PMC42, have been proven (19).

Microencapsulation is an emerging way to create drug nanocarriers and has provided reasonable explanations for drug delivery problems (23). Today, a large number of nanocarriers are used to transport therapeutic molecules (24, 25). Chitosan is a linear and biodegradable amino polysaccharide, which is environment-friendly and safe and increases the stability of bioactive compounds (26). Chitosan and its varied chemical structures, such as carboxymethyl chitosan, are known as important drug nanocarriers (27). Despite the remarkable success of the chemotherapy drug imatinib, one obstacle in prolonged therapy is the development of resistance mutations within the kinase domain of its target, Abl, in the last decades. The synergistic effects of imatinib and other natural agents on BCR-ABL positive imatinib-resistant K562 cells are not well studied, and there are rare investigations in this background. This study aimed to investigate the synergistic effects of quercetin and imatinib combination encapsulated with chitosan nanoparticles (Cs) on cytotoxicity, apoptosis, and K562 cell line growth.

## Materials and Methods

***Preparation of Cs containing imatinib and quercetin***

Cs were prepared by the ionic gelation method, according to the previous study (28). First, one gram of low molecular weight chitosan (Sigma) was completely dissolved in 50 ml of 1% acetic acid and stored at 100 rpm magnetic stirring for 5 hr at 25 °C. Then, 0.5 g of quercetin (Sigma) and imatinib mesylate (Merck) were added separately to the chitosan solution and mixed for 60 min. Then, 0.2 g of sodium tripolyphosphate was dissolved in 20 ml of deionized water and added to the previous solutions. After 60 min of mixing at room temperature at 13,000 rpm, it was centrifuged for 15 min, and the supernatant was evacuated. The Cs containing imatinib (Cs-IM) and quercetin (Cs-Qu) were then dried at 40 °C. The characteristics of nanoparticles were prepared by scanning electron microscope (SEM) imaging and Fourier-transform infrared spectroscopy (FTIR) method (Shimadzu, Kyoto, Japan). Surface charge (zeta potential), size distribution, scattering index (PDI), and the average size of Cs loaded were determined by Nano-ZS ZEN 3600 (Malvern Instruments Ltd, England) using the dynamic light scattering (DLS) technique. The encapsulation efficiency of the nanoparticles was determined by Cary 6000i UV-Vis-NIR Spectrophotometer (Agilent Technologies) at 370 nm λmax. 

***Cell culture***

K562 BCR-ABL positive cells (Bcr-Abl+ CML) were prepared from the Pasteur Institute of Iran. The cells were stored in RPMI1640 (Invitrogen) culture medium supplemented with 10% fetal bovine serum (FBS) (Life Technologies, USA) and 100 units/ml streptomycin sulfate. They were incubated at 37 °C and 5% CO_2_ and passaged every two days to maintain logarithmic growth. Imatinib-resistant K562 cells were developed by exposing the cells to increasing concentrations of imatinib (0.1 to 1M) (12).

***Cytotoxicity of drugs, nano drugs, and their combination***

The cytotoxicity of each drug in the natural and the encapsulated form and in combination with each other was determined by the standard MTT (3-[4,5-dimethylthiazol-2-yl]-2,5 diphenyl tetrazolium bromide) assay. Briefly, 8000 cells/well density of K562 cells were seeded in 96-well microtiter plates, and immediately, all drug treatments were added to the wells. Free and nano-encapsulated forms of quercetin and imatinib were added to wells containing cell suspension to evaluate the synergistic effect of drugs. Then MTT method was performed, absorption was measured at 570 nm, and the IC_50_ values ​​of drugs and their combinations were calculated. The combination index (CI) was calculated to determine the effect of the combination of drugs and nano drugs based on the IC_50_ values ​​of each drug alone and in combination with each other in Compusyn software (ComboSyn).

Using the formula CI = (D) 1 / (Dx) 1 + (D) 2 / (Dx) 2, CI was calculated where (Dx) 1 and (Dx) 2 represent the individual doses of free drugs or drugs-loaded Cs required to inhibit a given level of K562 cells growth, and (D)1 and (D)2 are the doses of free drugs or drug-loaded Cs necessary to produce the equal effect in combination, respectively. CI values >1 were considered as the synergistic effect of the agents.

***Evaluation of induction of apoptosis in K562 cells***

Annexin V-FITC (BD Bioscience, USA) apoptosis diagnosis kit was used to measure apoptosis. In summary, 3×10^5^ K562 cells were treated with RPMI (negative control) and IC_50_ concentrations of drugs and nano drugs for 48 hr. At the end of the treatment, cells were collected and washed with ice-cold phosphate-buffered saline (PBS), and the Annexin V-FITC staining procedure was performed according to kit instructions. The cytoFLEX S flow cytometer (Beckman, USA) and Cell Quest analysis software (BD Biosciences, USA) were used to analyze the samples.

In addition, DAPI (4′,6-diamidino-2-phenylindole) nuclei staining (Thermo Fisher Scientific, USA) was used to evaluate the morphological changes in apoptotic K562 cells (29). Briefly, 2×10^5^ K562 cells were seeded in a 96-well plate, and the culture medium supplemented with 10% FBS in each well was added to a final volume of 2 ml and kept in a CO_2_ incubator for 24 hr. Then, the culture medium was replaced with fresh culture medium and IC_50_ concentration of drugs, and their combination was added to the wells and plate incubated for 16 hr. Next, cells were collected in separate tubes and centrifuged. Then 0.5 ml of DAPI stain (1 mg/ml) was added to each tube and centrifuged. The supernatant was removed, and 0.5 ml of DAPI dye was added to the samples; then, the samples were incubated for 30 min in the dark. The cells were washed with cold PBS buffer and observed under an inverted fluorescence microscope (Olympus, Japan).

***Investigation of gene expression***

In this study, the effect of drugs on the expression of pro-apoptotic genes, Caspase-3, Caspase-8, and *TP53, *was investigated by real-time PCR. *GAPDH* was used as the housekeeping gene. Briefly, a total of 50,000 K562 cells were cultured in a 9-well plate with incubation conditions of 37 °C, 95% humidity, and 5% CO_2_. After cell adhesion for about 12 hr, cell culture was changed, IC_50_ concentrations of nano drugs and their compounds were added, and cells were collected 24 and 48 hr after drug exposure. The forward and reverse primers used for PCR amplification of *TP53*, Caspase-3, Caspase-8, and *GAPDH* genes were reported previously (30, 31). Total RNA was isolated with the RNX-PLUS kit (CinnaGen, Iran). The concentration and purity of RNA were evaluated by NanoDrop 1000 Spectrophotometer (NanoDrop Technologies, Wilmington, DE, USA); then, cDNA was synthesized using the RevertAid First Strand cDNA Synthesis Kit (Thermo Scientific). Finally, the expression of these genes was evaluated by Real-time PCR technique (Eppendorf, Germany) using the Power SYBR Green Master MIX kit. 

***Statistical analysis***

SPSS version 18 and Graphpad Prism version 6 software were used to perform the statistical analysis and plotting. The Kolmogorov-Smirnov test was used to evaluate the normality of data frequency distribution. The homogeneity of variances was determined by Levene’s test (*P*<0.05). Laboratory results were reported as mean ± standard deviation (in three replications). To evaluate the differences between the effects of different forms of the drug on the survival of cancer cells, a one-way analysis of variance was used at a significance level of 0.05, and Tukey’s honestly significant difference test was used.

## Results

***Nano drugs characterization***

**Figure 1:**
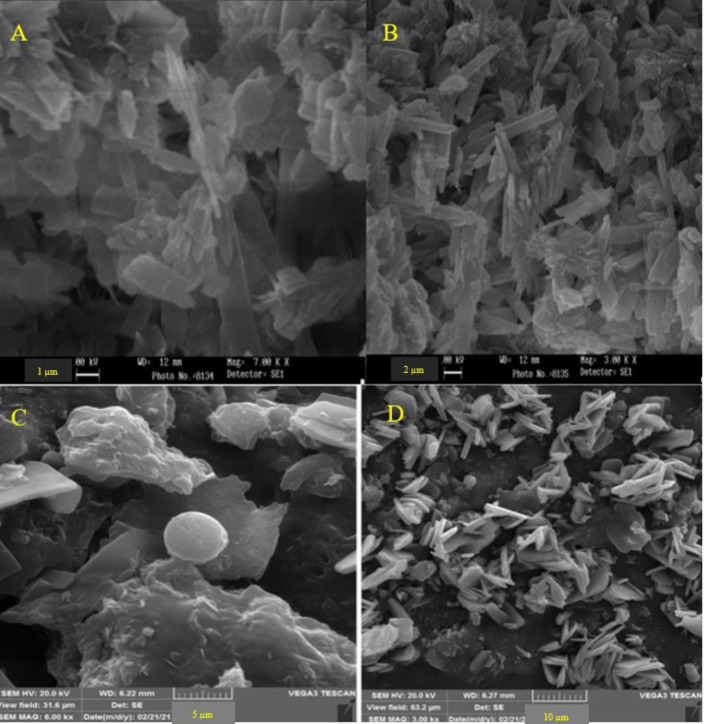
Scanning electron microscope image of quercetin nanoparticles (A, B) and imatinib nanoparticles (C, D) encapsulated with chitosan

**Figure 2:**
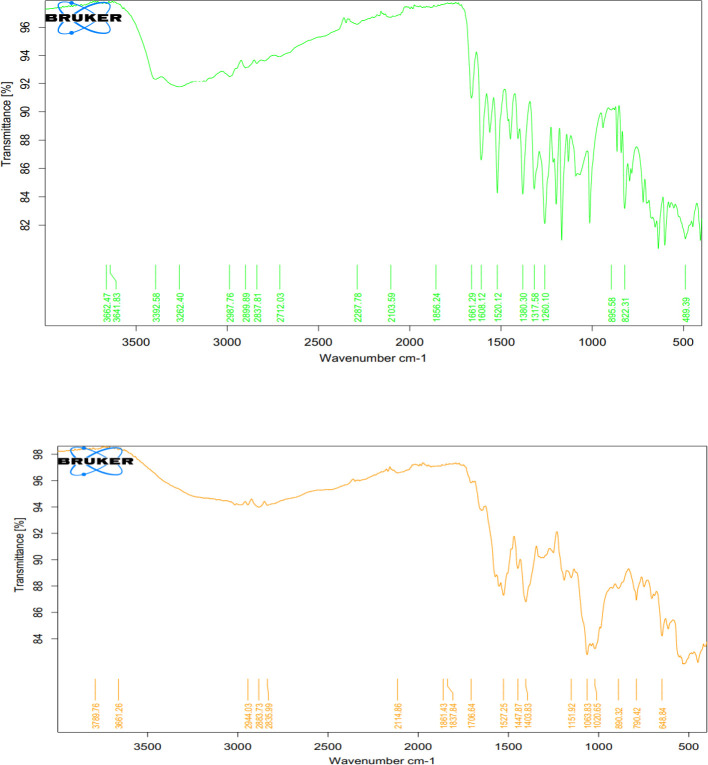
FTIR plot of Cs-Qu (above) and Cs-Im encapsulated with Cs

Examination of the data obtained from the study of physical properties showed that the size of imatinib encapsulated with Cs was from 170 nm to 244.8 nm (averages 205.6 nm), and the PDI in these nanoparticles was 0.129± 0.03. Also, the size of quercetin encapsulated with Cs was from 10 nm to 412 nm (average 156.3 nm), and the PDI in these nanoparticles was 0.462± 0.01 (Figure 1). The zeta potential of Cs-Im and Cs-Qu nanoparticles was +48.3 mV and +58.6 mV, respectively. The encapsulation efficacy for Cs-Im and Cs-Qu nanoparticles were 88.6 ± 0.2% and 91.1 ± 0.5%, respectively. Figure 2 shows the results of the FTIR study in chitosan-encapsulated imatinib and quercetin.

***Determination of IC***_50_ ***dose of the drugs***

**Figure 3:**
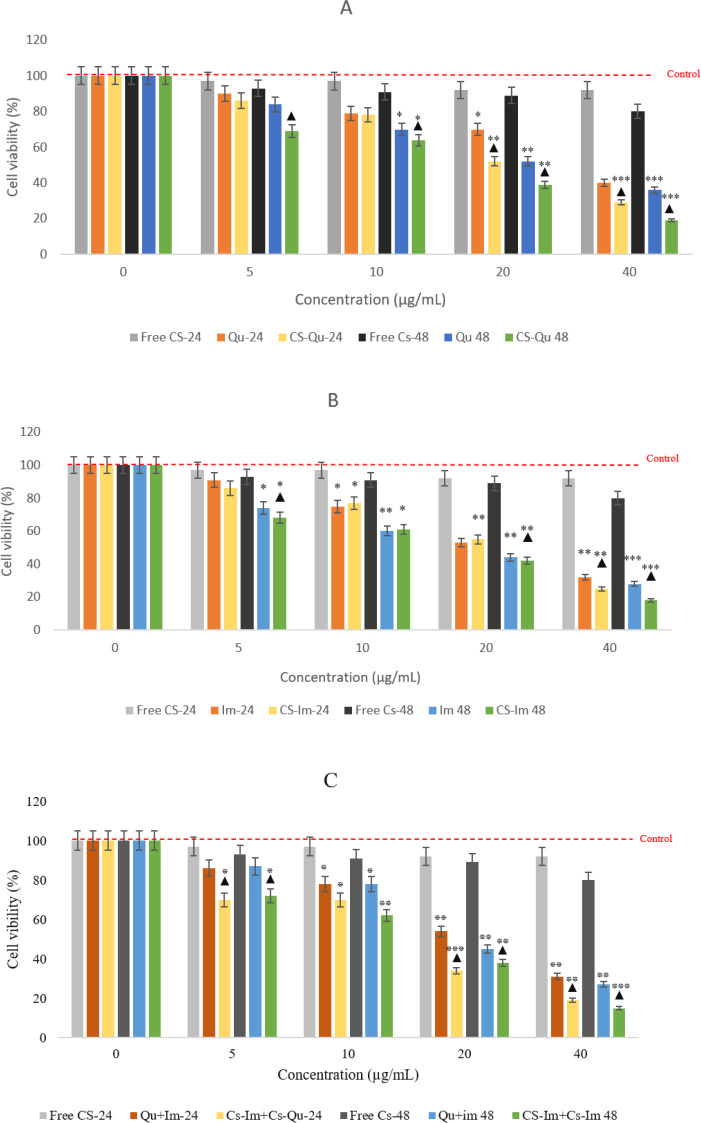
Cytotoxicity of formulations against imatinib-resistant BCR-ABL positive K562 cells in 96-well plates determined using the MTT assay. Relative cell viability following 24- and 48-hr treatment with IC_50_ values of free chitosan nanoparticles (free-Cs), free quercetin (Qu), and chitosan encapsulated quercetin (Cs-Qu) (A), free Imatinib (Im) and chitosan loaded imatinib (Cs-Im) (B), and in combination (C). Data presented as mean values ± standard deviation for triplicates. *** *P*<0.001, ** *P*<0.01 and *P<0.05 compared with control; ▲ *P*<0.01 vs. non-encapsulated drugs

The IC_50_ level during 24 hr of cell exposure was 23.85 μg/ml for free quercetin and 27.17 μg /ml for free imatinib. The IC_50_ level was 19.71 μg/ml for quercetin and 19.63 μg/ml for imatinib during 48 hr of treatment. In addition, the IC_50_ value for the combination of free quercetin and free imatinib was 12.35 μg/ml in 24 hr and 8.390 μg/ml in 48 hr. It should be noted that Cs-Qu and Cs-Im exhibited the IC_50_ values of 16.71 μg/ml and 13.28 μg/ml for 24 hr of treatment, respectively. The IC_50_ value for Cs-Qu and Cs-Im after 48 hr of treatment was 10.36 μg/ml and 10.86 μg/ml, respectively. In addition, the IC_50 _for combined Cs-Qu+ Cs-Im was reduced to 9.324 μg/ml in 24 hr and 6.68 μg/ml in 48 hr treatment. These findings demonstrate that the drugs, when encapsulated in Cs, showed cytotoxicity at lower concentrations compared to the free drug (Figure 3). The IC_50_ level during 24 and 48 hr of cell exposure was 96.6 μg/ml and 94.0 μg/ml for free Cs, respectively. 

***Synergistic effect***

**Figure 4:**
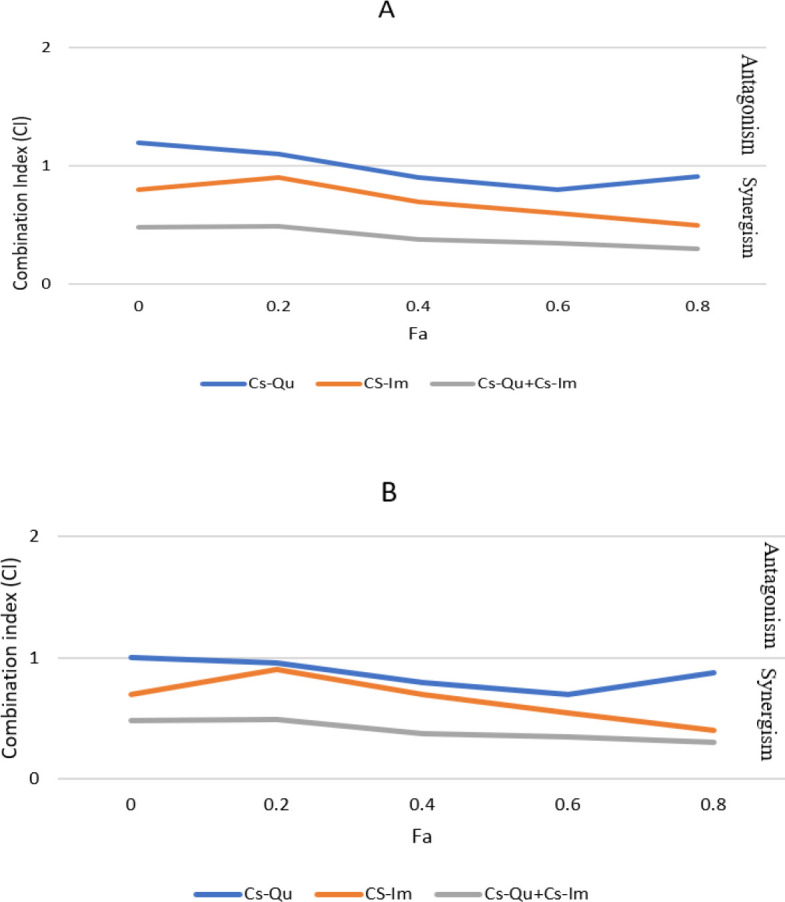
Combination index (CI) analysis. Fraction affected (Fa) versus CI plots were generated using Compusyn software to determine the extent of synergy for either chitosan-loaded quercetin and imatinib in combination with each other in imatinib-resistant BCR-ABL positive K562 cell line after 24 (A) and 48 hr (B) treatment. Additive effects were defined as CI=1, antagonistic effects are CI>1 and synergistic effects are CI<1

Examination of MTT outcomes to evaluate the survival of K562 cells revealed that the survival of cancer cells due to 48 hr and 24 hr drug treatment in all experimental groups was significantly lower than the control group (*P*<0.05). In addition, the survival rate in the cells exposed to encapsulated quercetin and imatinib was lower than the free forms of the drug (*P*<0.001). Furthermore, Compusyn software was used to identify the synergistic effect of the combination. The data showed that after cotreatment of cells with Cs-Qu and Cs-Im, the survival rate of K562 cells decreased compared to free drug and chitosan nanoparticles loaded alone treatment (*P*<0.001), (Figure 4) All CI values calculated by Compusyn software for the Cs-Qu+Cs-Im study were <0.5, suggesting that the growth inhibition influence of this combination in the K562 cells was synergistic rather than additive.

***Apoptosis induction***

**Figure 5:**
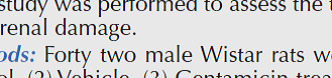
(A) K562 cells treated with Cs-Qu, Cs-Im, and their combination for 48 hours were stained with DAPI (Left). The relative fluorescence intensity analysis of DAPI was evaluated. The yellow arrows indicate the fragmented nuclei and DNA of the cells. (B) Images show the results of induction of apoptosis in K562 cells by staining with Annexin V staining kit in flow cytometry due to exposure to IC_50_ concentration of quercetin, imatinib, nano-quercetin, nano-imatinib, and their combination. The chart shows the cell apoptosis rate in K562 cells treated with nano-quercetin, nano-imatinib, and their combination. Data presented as mean values ± standard deviation for triplicates. Significant as compared to control (free chitosan): ***P<0.001, **P<0.01

Cs-Qu: Chitosan nano encapsulated quercetin; Cs-Im: Chitosan nano encapsulated imatinib mesylate

DAPI staining and evaluation of the cells in fluorescence microscopy confirmed the cytotoxic and anti-proliferative effects of IC_50_ concentrations of drugs and their combination. Microscopic examination clearly shows a decrease in cell number and morphological changes such as shrinkage, rounding, chromatin fragmentation, membrane protrusion, as well as fluorescent blue color and the formation of apoptotic bodies in treated K562 cells compared to the control cells (treated with free chitosan). This finding confirmed the inhibition of cell proliferation and induction of cell death in a dose-dependent manner (Figure 5a). In addition, flow cytometry showed that the drug combination of Cs-Qu+Cs-Im significantly increased apoptotic cells in comparison with cells treated with the free form of drugs (Figure 5b).

***Apoptotic genes expression***

**Figure 6:**
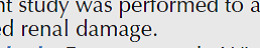
Fold changes in the target genes' mRNA expression levels relative to the housekeeping gene in the K562 cells treated with Cs-Qu, Cs-Im, and their combination. Significant as compared to housekeeping gene expression: * *P*<0.05, ** *P*<0.01, and *** *P*<0.001

Cs-Qu: Chitosan nano encapsulated quercetin; Cs-Im: Chitosan nano encapsulated imatinib mesylate

The cycle threshold analysis results of the real-time PCR reactions were evaluated using REST 2018 software before and after exposure to nanoquercetin, nanoimatinib, and their combination. The data demonstrated that the mean level of changes in gene expression was statistically significant. In the case of Cs-Im, the expression of caspase 3, 8, and *TP53* genes were upregulated by + 1.94, + 2.61, and + 2.11, respectively, in comparison with the *GAPDH* gene (*P*<0.001). The expression level of the caspase 3 gene was upregulated significantly after treatment with Cs-Qu (*P*<0.01). In addition, the increase in expression of all genes due to treatment with a combination Cs-Qu + CS-Im was statistically significant (*P*<0.001). (Figure 6). 

Cs: Chitosan nanoparticles; Cs-IM: Chitosan nanoparticles containing imatinib; Cs-Qu: Chitosan nanoparticles containing quercetin

## Discussion

It is now clear that drug regimens for refractory cancers are not very effective and, on the other hand, have severe side effects and even carcinogenesis, so the development of new treatments can be considered as a solution to treat these conditions (32). Investigations have revealed that delivering the drug to the target tissue, the so-called target therapy, can be a way to prevent drug resistance and decrease the side effects of chemotherapy medications (33). The results of the present study showed that the form of nano drugs encapsulated with chitosan was more effective than the free form of the drugs. In addition, the synergistic effect of the combination of encapsulated imatinib and quercetin was proved in statistical analysis. Marselin *et al.* investigated the effect of nano-drug delivery on reducing the side effects of imatinib mesylate. Imatinib-mesylate loaded poly lactide-co-glycolide nanoparticles were fabricated that could release the drug stably. Then, their effect was studied using *in vitro* anti-cancer methods and *in vivo* cardiac toxicity. The MTT assay showed that nano drugs were more cytotoxic to MCF-7 cancer cells than the equivalent concentration of free imatinib. Wistar rats treated orally with 50 mg/kg nano-drug for 28 days did not show cardiac toxicity or related changes. Increased alanine aminotransferase, aspartate aminotransferase, alkaline phosphatase, and decreased white blood cell, red blood cell, and hemoglobin levels were observed in animals treated with pure drugs. Based on these results, it was concluded that nanoencapsulation of imatinib mesylate increases its effect against cancer cells, almost without cardiac toxicity (34). In line with these findings, in the present study, MTT test data showed that encapsulation of imatinib mesylate increases its effectiveness compared to the free form of the drug.

In a study by Khakrizi *et al.*, the anti-cancer properties of the magnetic drug chitosan-hydroxyurea on the Hela cell line were investigated. Their results indicated that increasing the concentration of hydroxyurea-loaded nanoparticles decreases cell viability in a dose-dependent manner. The nano-drug also significantly raised the induction of apoptosis in treated HeLa cells by 2.48 times compared to the control group (35). These findings are in line with the findings of the present study on the effect of Cs on enhanced drug delivery to cancer cells. Both studies showed that the imatinib and hydroxyurea-loaded nanoparticles significantly reduced cell viability in a dose-dependent manner. In a study consistent with our results by Feng *et al.* (36), the capacity of the polyelectrolyte complex (CS / CMCS-NPs), consisting of chitosan and o-carboxymethitosan (CMCS) as a pH-responsive carrier for oral delivery of doxorubicin hydrochloride (DOX) was examined. Their study showed that the administration of DOX by the mentioned nanocarriers increases the stability of the drug in the blood. In addition, based on biopsy studies performed on the tissues of mice treated with synthetic nano drugs, these nanocarriers significantly reduced the toxicity of drugs on kidney and heart tissues (36). Hou *et al.* demonstrated that chitosan-conjugated folic acid and methoxy polyethylene glycol nanoparticles (mPEG-FA-CNP) were more adsorbed by tumor cells than by mPEG-CNP. This increased the toxicity of mitomycin C and the shelf life of nanoparticles in the blood (37), which is in line with our findings. Another study has shown that Cs at 37 °C protect against enzymes and cause the nanoparticle to bind effectively to the surface of human Bcap37 breast cancer cells (38). These data confirm the role of chitosan nanocarriers in increasing the cytotoxic nature of anticancer drugs, like the results of our research. In addition, it has been shown that chitosan is a good candidate for siRNA transmission, which has been very low and stable in blood plasma (39).

In another similar study, Shokrzadeh *et al.* evaluated the cytotoxicity of Docetaxel-loaded chitosan nanoparticles (DTX) in human liver cancer cells (40). Their results showed strong and concentration-dependent inhibition of HepG2 cancer cell proliferation by nanoparticles compared to free drugs. The outcomes of their study in accordance with the present study show that the nano-drug form can significantly increase the toxicity of the drug. Numerous studies have revealed that drug nanoparticles produce the right amount of drug, which leads to increased penetration into the intestinal mucosa as well as increased enhanced permeability and retention (EPR) (41). Research has shown that particles larger than 500 nanometers are much less absorbed in the gut than nanoparticles (42). In addition, the zeta potential of imatinib and quercetin nanoparticles was positive and showed a suitable and stable dispersion in the present study. This leads to increased absorption in the intestine and cells because the presence of chitosan particles with a positive charge leads to the electrostatic binding of nanoparticles to the innermost layer of the gastrointestinal tract with a negative charge (43). Other data show that the molecular weight of chitosan is an important factor in increasing cell penetration and the immune system’s low response to drugs (44). The lower IC_50_ of nano drugs than the free form of quercetin and imatinib indicates the higher sensitivity of K562 cells to nano drugs, which could be attributed to the greater and better loading of nanopharmaceuticals on cancer cells and the sustained and controlled release of the drug.

Lu *et al.* have indicated that the proliferation inhibition and apoptosis induction between imatinib-resistant and imatinib-sensitive cell lines treated with 25 µl/ml of quercetin for 24 hr were similar. The data showed that quercetin could not alter BCR-ABL expression. But, the γ-H2AX expression was significantly increased, and JNK phosphorylation by quercetin was regulated in both cell lines. Finally, they concluded that the growth of imatinib-resistant cells could be inhibited by quercetin. In addition, They showed that cell apoptosis could be induced by quercetin, which may be associated with cell cycle arrest at G2/M. DNA damage and increased p-JNK regulation may be involved in these processes (45). In another study, it has been shown that the effect of quercetin on BCR-ABL positive CML cells could be induced by affecting the Wnt/β-Catenin signaling pathway, leading to apoptosis in the K562 cell line. They showed that, following treatment with quercetin, mRNA and protein expression of caspase-3, 8, and 9, p21 and p27 increased in K562 cells compared to the control (46). Our findings demonstrated a significant increase in the mRNA expression of pro-apoptotic genes. In both studies, increased expression of caspase and p53 genes was observed, which indicates the mechanism by which quercetin affects K562 cells. On the other hand, this mechanism can be described as a justification for the significant synergistic effect of the combination of Cs-Im and Cs-Qu, as they both induce apoptosis through different signaling pathways in this cell line. In addition to the mechanisms mentioned, other studies have stated the effectiveness of quercetin in inducing the process of cell cycle arrest by affecting heat shock proteins and other epigenetic factors, which is different from the mechanism of action of imatinib (47). The synergistic effects of the two drugs used in this study can be attributed to different mechanisms of their action.

## Conclusion

The data of the present study show that the form containing chitosan imatinib and quercetin has more cytotoxicity than the free drug. On the other hand, the results showed that the combination of imatinib and quercetin as a nano-drug complex has a synergistic effect on the induction of apoptosis in imatinib-resistant BCR-ABL positive chronic human myeloid leukemia cells. Therefore, the combination of these drugs for treating refractory chronic myeloid leukemia could be further investigated.

## Authors’ Contributions

RK and AJ designed the experiments; NP performed experiments and collected data; RK and AJ discussed the results and strategy; RK and AJ supervised, directed, and managed the study; NP approved the final version to be published.

## Conflicts of Interestn

The authors declare no conflict of interest.

## References

[1] Siegel RL, Miller KD, Fuchs HE, Jemal A (2022). Cancer statistics, 2022. CA Cancer J Clin.

[2] Fernandes A, Shanmuganathan N, Branford S (2022). Genomic mechanisms influencing outcome in chronic myeloid leukemia. Cancers (Basel).

[3] Sumi K, Tago K, Nakazawa Y, Takahashi K, Ohe T, Mashino T (2022). Novel mechanism by a bis-pyridinium fullerene derivative to induce apoptosis by enhancing the MEK-ERK pathway in a reactive oxygen species-independent manner in BCR-ABL-positive chronic myeloid leukemia-derived K562 cells. Int J Mol Sci.

[4] Sampaio MM, Santos MLC, Marques HS, Goncalves VLS, Araujo GRL, Lopes LW (2021). Chronic myeloid leukemia-from the Philadelphia chromosome to specific target drugs: A literature review. World J Clin Oncol.

[5] Amarante-Mendes GP, Rana A, Datoguia TS, Hamerschlak N, Brumatti G (2022). BCR-ABL1 tyrosine kinase complex signaling transduction: challenges to overcome resistance in chronic myeloid leukemia. Pharmaceutics.

[6] Kayabasi C, Caner A, Yilmaz Susluer S, Balci Okcanoglu T, Ozmen Yelken B, Asik A (2022). Comparative expression analysis of dasatinib and ponatinib-regulated lncRNAs in chronic myeloid leukemia and their network analysis. Med Oncol.

[7] De Santis S, Monaldi C, Mancini M, Bruno S, Cavo M, Soverini S (2022). Overcoming resistance to kinase inhibitors: the paradigm of chronic myeloid leukemia. Onco Targets Ther.

[8] Chandrasekhar C, Kumar PS, Sarma P (2019). Novel mutations in the kinase domain of BCR-ABL gene causing imatinib resistance in chronic myeloid leukemia patients. Sci Rep.

[9] Jang B, Kwon H, Katila P, Lee SJ, Lee H (2016). Dual delivery of biological therapeutics for multimodal and synergistic cancer therapies. Adv Drug Deliv Rev.

[10] Wang S, Liu X, Wang S, Ouyang L, Li H, Ding J (2021). Imatinib co-loaded targeted realgar nanocrystal for synergistic therapy of chronic myeloid leukemia. J Control Release.

[11] Oaxaca DM, Yang-Reid SA, Ross JA, Rodriguez G, Staniswalis JG, Kirken RA (2016). Sensitivity of imatinib-resistant T315I BCR-ABL CML to a synergistic combination of ponatinib and forskolin treatment. Tumour Biol.

[12] Wei Y, To KK, Au-Yeung SC (2015). Synergistic cytotoxicity from combination of imatinib and platinum-based anticancer drugs specifically in Bcr-Abl positive leukemia cells. J Pharmacol Sci.

[13] Zhou W, Zhu W, Ma L, Xiao F, Qian W (2015). Proteasome inhibitor MG-132 enhances histone deacetylase inhibitor SAHA-induced cell death of chronic myeloid leukemia cells by an ROS-mediated mechanism and downregulation of the Bcr-Abl fusion protein. Oncol Lett.

[14] La Rosee P, O’Dwyer M, Druker B (2002). Insights from pre-clinical studies for new combination treatment regimens with the Bcr-Abl kinase inhibitor imatinib mesylate (Gleevec/Glivec) in chronic myelogenous leukemia: a translational perspective. Leukemia.

[15] Dharmapuri G, Doneti R, Philip GH, Kalle AM (2015). Celecoxib sensitizes imatinib-resistant K562 cells to imatinib by inhibiting MRP1-5, ABCA2 and ABCG2 transporters via Wnt and Ras signaling pathways. Leuk Res.

[16] Jin Y, Yao Y, Chen L, Zhu X, Jin B, Shen Y (2016). Depletion of γ-catenin by histone deacetylase inhibition confers elimination of CML stem cells in combination with imatinib. Theranostics.

[17] Tang SM, Deng XT, Zhou J, Li QP, Ge XX, Miao L (2020). Pharmacological basis and new insights of quercetin action in respect to its anti-cancer effects. Biomed Pharmacother.

[18] Reyes-Farias M, Carrasco-Pozo C (2019). The anti-cancer effect of quercetin: molecular implications in cancer metabolism. Int J Mol Sci.

[19] Vafadar A, Shabaninejad Z, Movahedpour A, Fallahi F, Taghavipour M, Ghasemi Y (2020). Quercetin and cancer: new insights into its therapeutic effects on ovarian cancer cells. Cell Biosci.

[20] Hashemzaei M, Delarami Far A, Yari A, Heravi RE, Tabrizian K, Taghdisi SM (2017). Anticancer and apoptosisinducing effects of quercetin in vitro and in vivo. Oncol Rep.

[21] Ren KW, Li YH, Wu G, Ren JZ, Lu HB, Li ZM (2017). Quercetin nanoparticles display antitumor activity via proliferation inhibition and apoptosis induction in liver cancer cells. Int J Oncol.

[22] Srivastava NS, Srivastava RAK (2019). Curcumin and quercetin synergistically inhibit cancer cell proliferation in multiple cancer cells and modulate Wnt/beta-catenin signaling and apoptotic pathways in A375 cells. Phytomedicine.

[23] Rahaiee S, Assadpour E, Faridi Esfanjani A, Silva AS, Jafari SM (2020). Application of nano/microencapsulated phenolic compounds against cancer. Adv Colloid Interface Sci.

[24] Barakat H (2020). Amygdalin as a plant-based bioactive constituent: a mini-review on intervention with gut microbiota, anticancer mechanisms, bioavailability, and microencapsulation. Proceedings.

[25] Avramovic N, Mandic B, Savic-Radojevic A, Simic T (2020). Polymeric nanocarriers of drug delivery systems in cancer therapy. Pharmaceutics.

[26] Shakeran Z, Keyhanfar M, Varshosaz J, Sutherland DS (2021). Biodegradable nanocarriers based on chitosan-modified mesoporous silica nanoparticles for delivery of methotrexate for application in breast cancer treatment. Mater Sci Eng C Mater Biol Appl.

[27] Hu Q, Luo Y (2021). Chitosan-based nanocarriers for encapsulation and delivery of curcumin: A review. Int J Biol Macromol.

[28] Agarwal M, Agarwal MK, Shrivastav N, Pandey S, Das R, Gaur P (2018). Preparation of chitosan nanoparticles and their in-vitro characterization. Int J Life Sci Scienti Res.

[29] Yang L, Li D, Tang P, Zuo Y (2020). Curcumin increases the sensitivity of K562/DOX cells to doxorubicin by targeting S100 calcium-binding protein A8 and P-glycoprotein. Oncol Lett.

[30] Mitupatum T, Aree K, Kittisenachai S, Roytrakul S, Puthong S, Kangsadalampai S (2016). mRNA expression of Bax, Bcl-2, p53, Cathepsin B, Caspase-3 and Caspase-9 in the HepG2 cell line following induction by a novel monoclonal Ab Hep88 mAb: cross-talk for paraptosis and apoptosis. Asian Pac J Cancer Prev.

[31] Zhou H, Zhou M, Hu Y, Limpanon Y, Ma Y, Huang P (2022). TNF-alpha triggers RIP1/FADD/Caspase-8-mediated apoptosis of astrocytes and RIP3/MLKL-mediated necroptosis of neurons induced by Angiostrongylus cantonensis infection. Cell Mol Neurobiol.

[32] Oien DB, Pathoulas CL, Ray U, Thirusangu P, Kalogera E, Shridhar V (2021). Repurposing quinacrine for treatment-refractory cancer. Semin Cancer Biol.

[33] Du W, Huang H, Sorrelle N, Brekken RA (2018). Sitravatinib potentiates immune checkpoint blockade in refractory cancer models. JCI Insight.

[34] Marslin G, Revina AM, Khandelwal VKM, Balakumar K, Prakash J, Franklin G (2015). Delivery as nanoparticles reduces imatinib mesylate-induced cardiotoxicity and improves anticancer activity. Int J Nanomedicine.

[35] Khakrizi E, BikhofTorbati M, Shaabanzadeh M (2018). The study of anticancer effect of magnetic chitosan-hydroxyurea nanodrug on HeLa cell line: a laboratory study. JRUMS.

[36] Feng C, Wang Z, Jiang C, Kong M, Zhou X, Li Y (2013). Chitosan/o-carboxymethyl chitosan nanoparticles for efficient and safe oral anticancer drug delivery: in vitro and in vivo evaluation. Int J Pharm.

[37] Hou Z, Zhan C, Jiang Q, Hu Q, Li L, Chang D (2011). Both FA-and mPEG-conjugated chitosan nanoparticles for targeted cellular uptake and enhanced tumor tissue distribution. Nanoscale Res Lett.

[38] Cao X, Chen C, Yu H, Wang P (2015). Horseradish peroxidase-encapsulated chitosan nanoparticles for enzyme-prodrug cancer therapy. Biotechnol Lett.

[39] Ragelle H, Riva R, Vandermeulen G, Naeye B, Pourcelle V, Le Duff CS (2014). Chitosan nanoparticles for siRNA delivery: optimizing formulation to increase stability and efficiency. J Control Release.

[40] Shokrzadeh M, Ebrahimnejad P, Omidi M, Shadboorestan A, Zaalzar Z (2012). Cytotoxity evaluation of docetaxel nanoparticles by culturing HepG2 carcinoma cell lines. J Mazandaran Univ Med Sci.

[41] Zhang R, Li Y, Zhou M, Wang C, Feng P, Miao W (2019). Photodynamic chitosan nano-assembly as a potent alternative candidate for combating antibiotic-resistant bacteria. ACS Appl Mater Interfaces.

[42] Sang M, Han L, Luo R, Qu W, Zheng F, Zhang K (2020). CD44 targeted redox-triggered self-assembly with magnetic enhanced EPR effects for effective amplification of gambogic acid to treat triple-negative breast cancer. Biomater Sci.

[43] Badran MM, Alomrani AH, Harisa GI, Ashour AE, Kumar A, Yassin AE (2018). Novel docetaxel chitosan-coated PLGA/PCL nanoparticles with magnified cytotoxicity and bioavailability. Biomed Pharmacother.

[44] Alomrani A, Badran M, Harisa GI, ALshehry M, Alhariri M, Alshamsan A (2019). The use of chitosan-coated flexible liposomes as a remarkable carrier to enhance the antitumor efficacy of 5-fluorouracil against colorectal cancer. Saudi Pharm J.

[45] Lu H-Y, Chen J, SH D, Jia P-M, Tong J-H, Wu Y-L (2017). Effects of quercetin on chronic myeloid leukemia cell line resistant to imatinib and its mechanism. Zhongguo Shi Yan Xue Ye Xue Za Zhi.

[46] Li W, Zhao Y, Qiu L, Ma J (2019). [Effect of quercetin on wnt/beta-catenin signal pathway of K562 and K562R cells]. Zhongguo Shi Yan Xue Ye Xue Za Zhi.

[47] Hassanzadeh A, Hosseinzadeh E, Rezapour S, Vahedi G, Haghnavaz N, Marofi F (2019). Quercetin promotes cell cycle arrest and apoptosis and attenuates the proliferation of human chronic myeloid leukemia cell line-K562 through interaction with HSPs (70 and 90), MAT2A and FOXM1. Anticancer Agents Med Chem.

